# Evaluation of indigenous entomopathogenic nematodes in Southwest China as potential biocontrol agents against *Spodoptera litura* (Lepidoptera: Noctuidae)

**DOI:** 10.21307/jofnem-2021-083

**Published:** 2021-11-22

**Authors:** Bingjiao Sun, Xiuqing Zhang, Li Song, Lixin Zheng, Xianqin Wei, Xinghui Gu, Yonghe Cui, Bin Hu, Toyoshi Yoshiga, Mahfouz M. Abd-Elgawad, Weibin Ruan

**Affiliations:** 1 College of Life Sciences, Nankai University, Tianjin, 30071, China; 2 Tianjin Recyclable Resources Institution, China Co-op, Tianjin, 300191, China; 3 Tobacco Company, Yuxi, 653100, Yunnan, China; 4 Beijing Plant Protection Station, Beijing, 100029, China; 5 Faculty of Agriculture, Saga University, Saga, 8408502, Japan; 6 Plant Pathology Department, National Research Centre, ElBehoos St., Dokki, Giza, 12622, Egypt

**Keywords:** *Heterorhabditis*, *Steinernema*, *Oscheius*, Phylogeny, Biocontrol

## Abstract

*Spodoptera litura* is a notorious leaf feeding insect pest in the Asia-Pacific region and leads to a significant economic loss in vegetable and field crop production. Entomopathogenic nematodes (EPNs), lethal parasites of insects, are used as biocontrol agents. Yunnan Province in China is a well-known region due to its rich biodiversity. In the present study, a survey of EPNs using the *Galleria*-baiting technique was conducted in 2017 and 2018 throughout the entire Yunnan province. In total, 789 soil samples were collected from 232 sites, of which 75 samples were positive for EPNs. Phylogenetic analyses of ITS, D2D3 expansion region of the 28S rRNA gene, as well as mitochondrial cytochrome c oxidase subunit I (COI), were performed to identify isolated nematode species and evaluate their genetic diversity. In total, 13, 3, and 58 identified populations belong to *Steinernema*, *Heterorhabditis*, and *Oscheius*, respectively. The phylogenetic relationships of EPN species in the three genera were analyzed with the Neighbor-Joining method. The virulence of the trapped isolates in the genera of *Steinernema*, *Heterorhabditis*, and *Oscheius* against *S. litura* was evaluated. Ten new indigenous isolates from *Steinernema* and *Heterorhabditis* showed prominent virulence to *S. litura* within 48 hr which is equivalent to that of commercial EPNs populations. The present study provides background information on indigenous EPN resources for *S. litura* control in Asia-Pacific region.

Entomopathogenic nematodes (EPNs) are obligate parasites of insect hosts and cause host mortality within 24–48 hr after infection (Griffin et al., 2005). To date, around 100 valid species of *Steinernema* and 21 species of *Heterorhabditis* have been identified from different countries of the world (Bhat and Askary, 2020). EPNs are promising candidates for biocontrol of insects due to their ability to search for hosts, safety to non-target organisms and the environment, high reproductive potential, ability to mass produce and compatibility with agricultural chemicals (Griffin et al., 2005). Persistence in the field can vary but has been documented up to 23 months or longer (Susurluk and Ehlers, 2008). More than a dozen EPN species in two families (Heterorhabditidae and Steinernematidae) have been commercialized for use in biological control (Shapiro-Ilan et al., 2018). Apart from *Heterorhabditis* and *Steinernema*, recently, several species from *Oscheius* were confirmed as EPNs (Castro-Ortega et al., 2020; Ye et al., 2018).

While developing a durable management strategy for inundative release of EPNs against local pest insects, indigenous nematodes are more suitable because of their adaptation to local climate and host population (Bedding, 1990). Thus, exploration of indigenous EPN is pivotal for EPN application in certain area; surveys conducted around the world demonstrated widespread occurrence and provided wide information on the indigenous species and populations of EPNs and their virulence to the target insect pest (Vashisth et al., 2017). For example, several commercial EPNs were tested to control *Popillia japonica* detected in Northern Italy in 2014 with variable results (Marianelli et al., 2018), and thus the dire need for locally and adapted populations of EPNs is well-recognized. Indigenous natural populations of *H. bacteriophora* have been shown to be more efficient in controlling *P. japonica* grubs (Torrini et al., 2020).

Yunnan Province located in southwest China has a vast territory with diverse and unique natural resources, and known as a biodiversity hotspot in the world (Myers et al., 2000). All these ranges are specific and rich in insect fauna and thus a potentially great opportunity for EPN recovery. However, the background information on EPNs across the region still remains unknown.

*Spodoptera litura* (Lepidoptera: Noctuidae) is a notorious leaf feeding insect pest attacking more than 120 different host plants (cotton, tobacco, maize, cabbage, groundnut, tea, etc.) around the Asia-Pacific region including Yunnan Province (https://gd.eppo.int/taxon/PRODLI/distribution), causing significant economic loss in vegetable and field crop production (Ahmad and Mehmood, 2015; Sang et al., 2016). *Spodoptera litura* has developed serious resistance to many traditional chemical insecticides (Yasin et al., 2020). As the application of harmful chemical pesticides affects the entire environment, there is an urgent need to develop eco-friendly approaches to control the pest. It was reported that EPN species, such as *Heterorhabditis indica*, *Steinernema carpocapsae*, and *S. longicaudum* can be used as efficient biological control agents against *S. litura* (Acharya et al., 2020; Lalramnghaki et al., 2020; Yan et al., 2020). However, the information on the virulence of indigenous EPN species in Yunan Province, China against *S. litura* is lacking.

To address the knowledge gap, we conducted a survey of the biodiversity and distribution of EPN fauna in Yunnan province by collecting 789 soil samples from 232 sites during 2017 and 2018. The major objectives of the present study were (i) to investigate the natural occurrence of indigenous EPNs in the entire Yunnan province by standard genotyping procedures (Al-Zaidawi et al., 2019; Edmunds et al., 2018), and (ii) to test the virulence of the detected indigenous EPNs isolates against *S. litura.*

## Materials and methods

### Collection sites and sampling method

Soil samples were collected from Yunnan province in southwest China during 2017–2018. Yunnan is at the far southern edge of the Tibet Plateau, with elevations that are the highest in the northwest (Kawagebo Peak, 6,740.0 m) and the lowest in the southeast (Red River Valley, 76.4 m) across approximately 8° of latitude from north to south. There are more than 18,000 higher plant species (51.6% of China’s total) and 1,836 vertebrate species (54.8% of China’s total) living in Yunnan on a land area of 39.4 × 104 km^2^, i.e., only 4.1% of China’s total (Yang et al., 2004).

**Figure 1:**
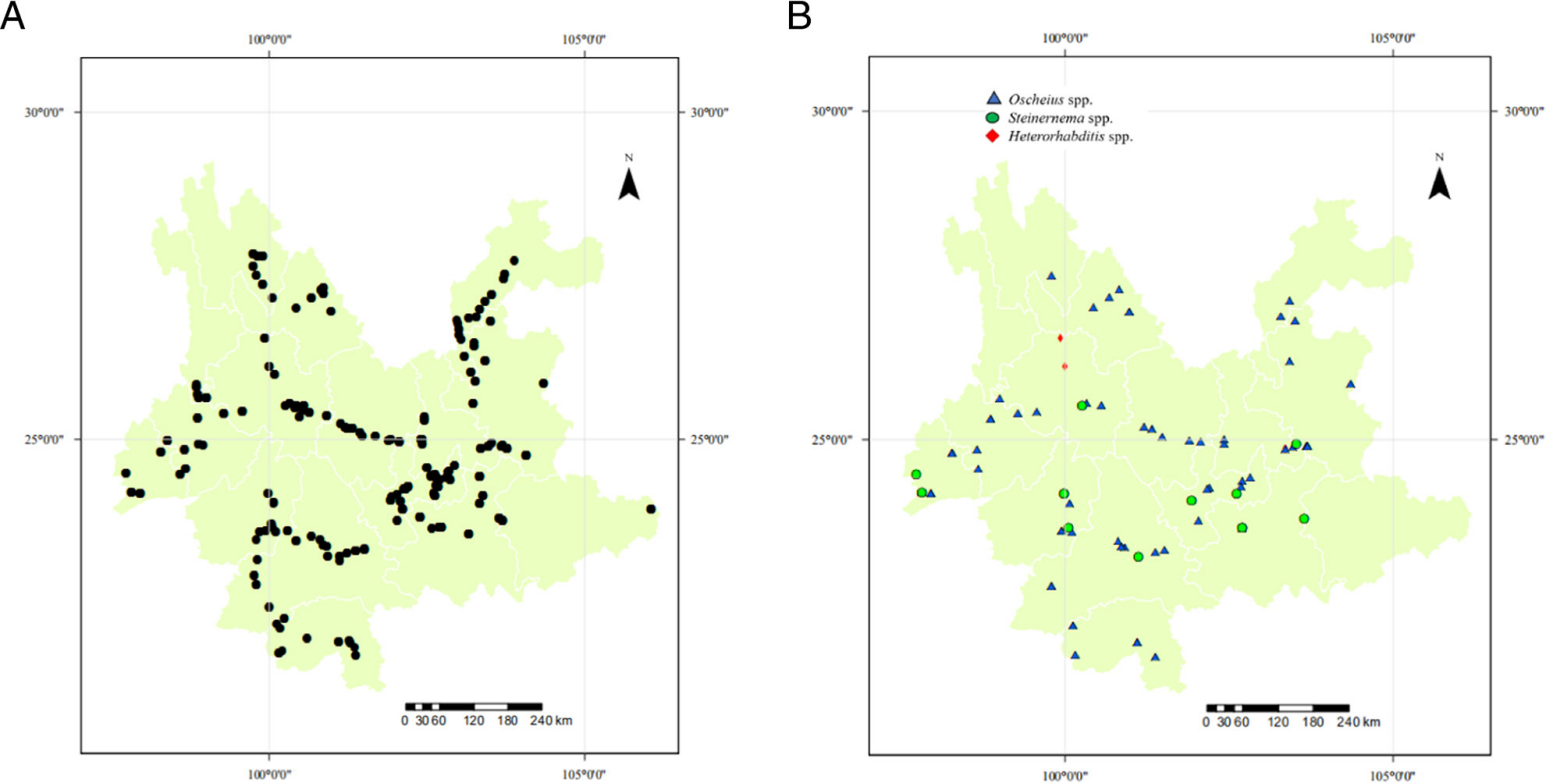
The distribution map of collected soil samples and entomopthogenic nematodes in Yunnan province, China. A: collected soil samples. B: entomopathogenic nematodes.

In total 789 soil samples were collected across Yunnan province (Fig. 1A). The collection sites were chosen to get a broad distribution across the entire Yunnan province. Each soil sample (approximately 500 g) was collected with a hand trowel to a depth of 0–15 cm. At the time of each collection, elevation, sampling date, associated vegetation, and GPS coordinates for each collection site were also recorded. To prevent moisture loss during transportation, the samples were placed separately in a polythene zipper bag, labeled and sent to the laboratory as soon as possible and subjected to EPNs trapping within two weeks.

### Soil physicochemical measurement

Soil pH was measured in a 1:2 soil-distilled H_2_O suspension using a glass electrode (Sartorius PB-10). Soil total carbon, total nitrogen and cation exchange capacity (CEC) were measured according to the method developed by Bao (2000). Data of the soil texture were provided by Data Center for Resources and Environmental Sciences, Chinese Academy of Sciences (RESDC) (http://www.resdc.cn).

### Isolation of EPNs with *Galleria mellonella*

Nematodes were recovered from the soil samples by using a modified insect-baiting technique (Bedding and Akhurst, 1975). Five last instar larvae of *G. mellonella* were placed in a steel tea leakage container filled with wet medical gauze, which was placed in a zipper bag containing soil samples. Soil samples were kept at room temperature (25 ± 2°C) for 2 weeks. The larvae were checked every two days and added to the bag with an appropriate amount of distilled water to maintain soil moisture if soil was really dry. Dead larvae were removed and transferred into modified White traps (White, 1927) for collection of IJs. These IJs were transferred into cell culture flasks and stored at 14°C. Koch’s postulates were performed to confirm the pathogenicity of the isolated EPNs (Kaya and Stock, 1997).

### Molecular identification of EPN species

Genomic DNA was extracted from the nematodes using a modified method (Stock et al., 2001). In total, 15 IJs nematodes were placed into a 200 μL tube containing 20 μL lysis buffer (50 mM KCl, 10 mM Tris pH 8.3, 2.5 mM MgCl_2_∙6H_2_O, 0.45% Nonidet P-40, 1% Trition X-100 and 60 μg/mL proteinase K). The mixture was incubated at 60°C for 1 hr and 95°C for 15 min in a thermocycler (Lissemore et al., 2005). Fragment of rDNA containing internal transcribed spacer (ITS1, 5.8S, ITS2) was amplified using primers 18S: 5′-TTG ATT ACG TCC CTG CCC TTT-3′ (forward), and 28S: 5′-TTT CAC TCG CCG TTA CTA AGG-3′ (reverse) (Vrain et al., 1992). The other fragment containing D2-D3 regions of 28S rDNA was amplified using primers D2F: 5′-CCTTAGTAACGGCGAGTGAAA-3′ (forward) and 536: 5′-CAGCTATCCTGAGGAAAC-3′ (reverse) (Nguyen and Hunt, 2007). COI-F1 (5′-CCTACTATGATTGGTGGTTTTGGTAATTG-3′) and COI-R2 (5′-GTAGCAGCAGTAAAATAAGCACG-3′) were used for amplification of the mitochondrial cytochrome oxidase c subunit1 (mtCOI) gene (Kanzaki and Futai, 2002). The Polymerase chain reactions (PCR) cycling profiles for the ITS, D2-D3 regions, and COI were the same as those described by Noujeim et al. (2017).

Each PCR reaction was made in a total volume of 25 μL containing 12.5 μL × Es Taq MasterMix (CWBIO, China) 0.5 μL 10 μM of each primer, 2 μL template DNA and 9.5 μL ddH_2_O. PCR products were electrophoresed in 1.5% agarose gel, purified and sequenced in both directions sequenced with ABI 3730 (Suzhou Genewiz Biotechnology Co., Ltd., Tianjin, China). Sequences were aligned using ClustalW with the default settings in MEGA X software package (Kumar et al., 2018). Sequences were visually proofread, edited, and assembled into contigs in Bioedit v7.1.7 (Hall, 2012). The above sequences were submitted to GenBank. Sequences of EPNs from Genbank were searched and involved into the phylogenetic analysis. Pairwise distances and Neighbor-Joining (NJ) (Saitou and Nei, 1987) phylogenetic analysis were done using MEGA X software package (Kumar et al., 2018) under a Kimura 2-parameter (Kimura, 1980) model. Bootstrap analysis was computed with 500 replicates.

### Virulence of EPNs against *S. litura* larvae

The virulence of the EPN isolates only in the genera of *Steinernema* and *Heterorhabditis* were evaluated for their virulence against *Spodoptera litura* (Lepidoptera: Noctuidae). The eggs of *S. litura* were purchased from Henan Jiyuan Baiyun Industry Co., Ltd. *Spodoptera* artificially reared in our laboratory at 25°C with a photoperiod of 12/12 (L/D) h and RH of 60–70% were used for virulence bioassay (Singh et al., 1983).

Virulence assays were performed as described by Zhen et al. (2018). Briefly, a total of 100 IJs in 30 μL and one 5th instar larva of *S. litura* were added to each well of a 24-well tissue culture plate lined with a filter paper. Each larva was placed in one well of a 24-well tissue culture plate. A total of five larvae per culture plate was treated as one replicate. Each treatment had five replicates containing 25 larvae. Sterile water (30 μL) was used as control. The control had seven replicates. The 24-well culture plates were sealed with Parafilm and placed into an incubator at 25°C, 60–70% RH in the dark. Number of dead larvae was recorded at 12 hr intervals for 84 hr. The virulence experiment was done twice in time. Besides the indigenous EPN isolates, two commercial EPNs, *S. carpocapsae* All and *H. bacteriophora* HbHb, were included as positive control, which were purchased from Zhejiang Lvshen Natural Enemy Biotechnology Co., Ltd, China. Infective juveniles (IJs) were collected on White traps at least 5 to 7 days after the start of emergence from *Gallaria mellonella* cadavers (White, 1927). IJs were rinsed with sterile water three times, and stored at 14°C and used within 3 weeks. The commercial populations were cultured in parallel before the assays to control of age of nematode.

### Data statistics

*Spodoptera litura* mortality were calculated as percentage. To evaluate the virulence of EPN isolates, one-way analysis of variance was conducted in SPSS 19.0. Differences among means were considered significant at *p* ≤ 0.05. The post hoc Duncan’s test was used to further elucidate treatment differences. Means ± SE are presented.

## Results

### Identification of EPNs from soil samples from Yunnan Province

We present a generalized evaluation of distribution of the entomopathogenic nematodes *Steinernema* spp., *Heterorhabditis* spp., and *Oscheius* spp. in different regions of Yunnan province. A total of 789 soil samples at 232 sites (Fig. 1A) were collected from different habitats including forest (287), cropland (249), grassland (99), waterside (34), and the edge of cropland (120) (Table 1).

**Table 1.** Distribution of entomopathogenic nematodes in different habitats in Yunnan Province, China.

	Forest	Farmland	Grassland	Waterside	The edge of cropland	Total
Soil sample No.	287	249	99	34	120	789
*Heterorhabditis* No.	0	3	0	0	0	3
*Steinernema* No.	7	3	1	1	1	13
*Oscheius* No.	35	10	5	4	4	58
Prevalence of *Heterorhabditis* and *Steinernema* (%)	2.44	2.41	1.01	2.94	0.83	2.03
Prevalence of *Oscheius* (%)	12.20	4.02	5.05	11.76	3.33	7.35

**Figure 2:**
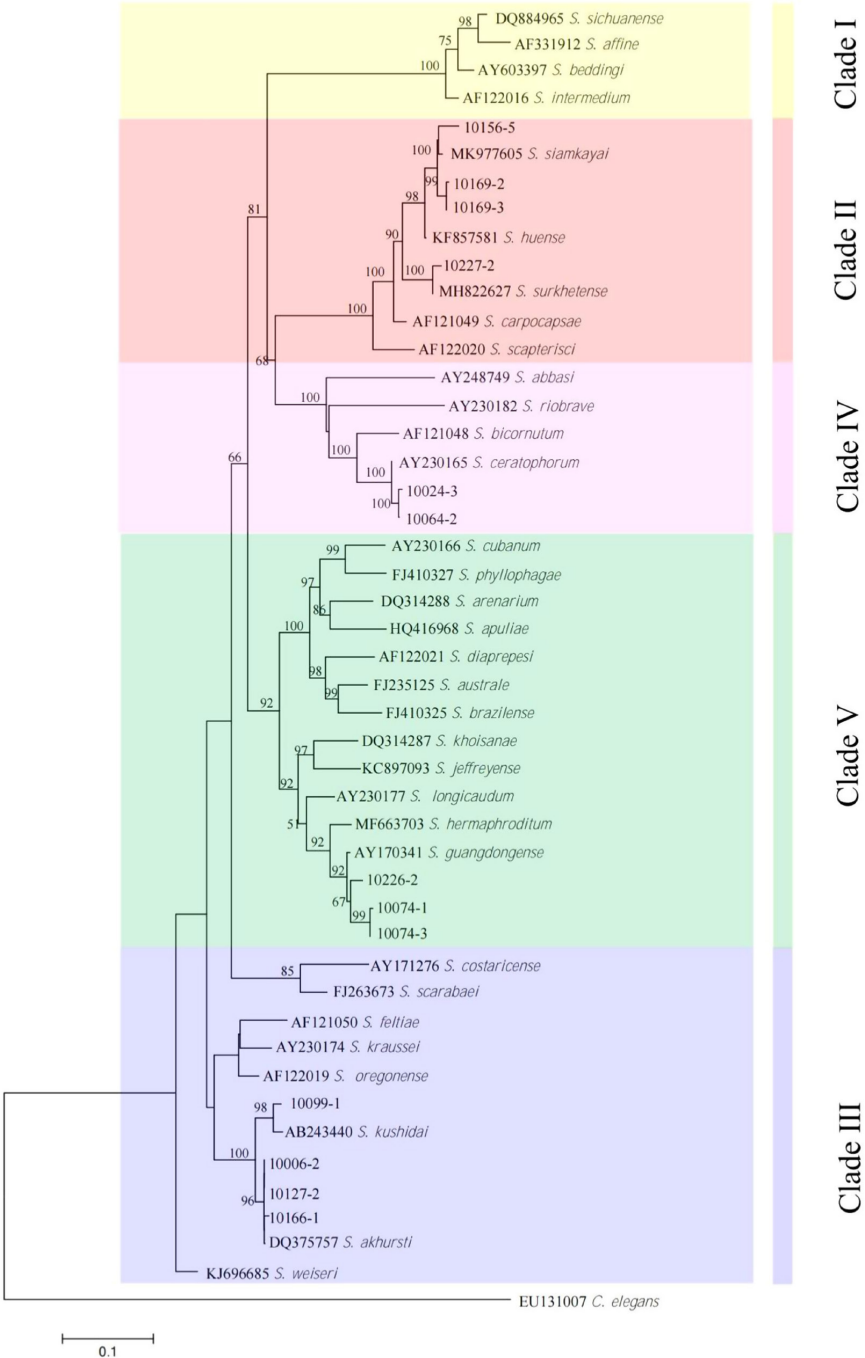
Neighbor-Joining tree of the *Steinernema* populations. The tree was based on ITS rDNA data and Kimura 2-parameter model. Numbers on branches represent bootstrap support (>50%) based on 1,000 replicates. Scale represents K2P genetic distance.

**Figure 3:**
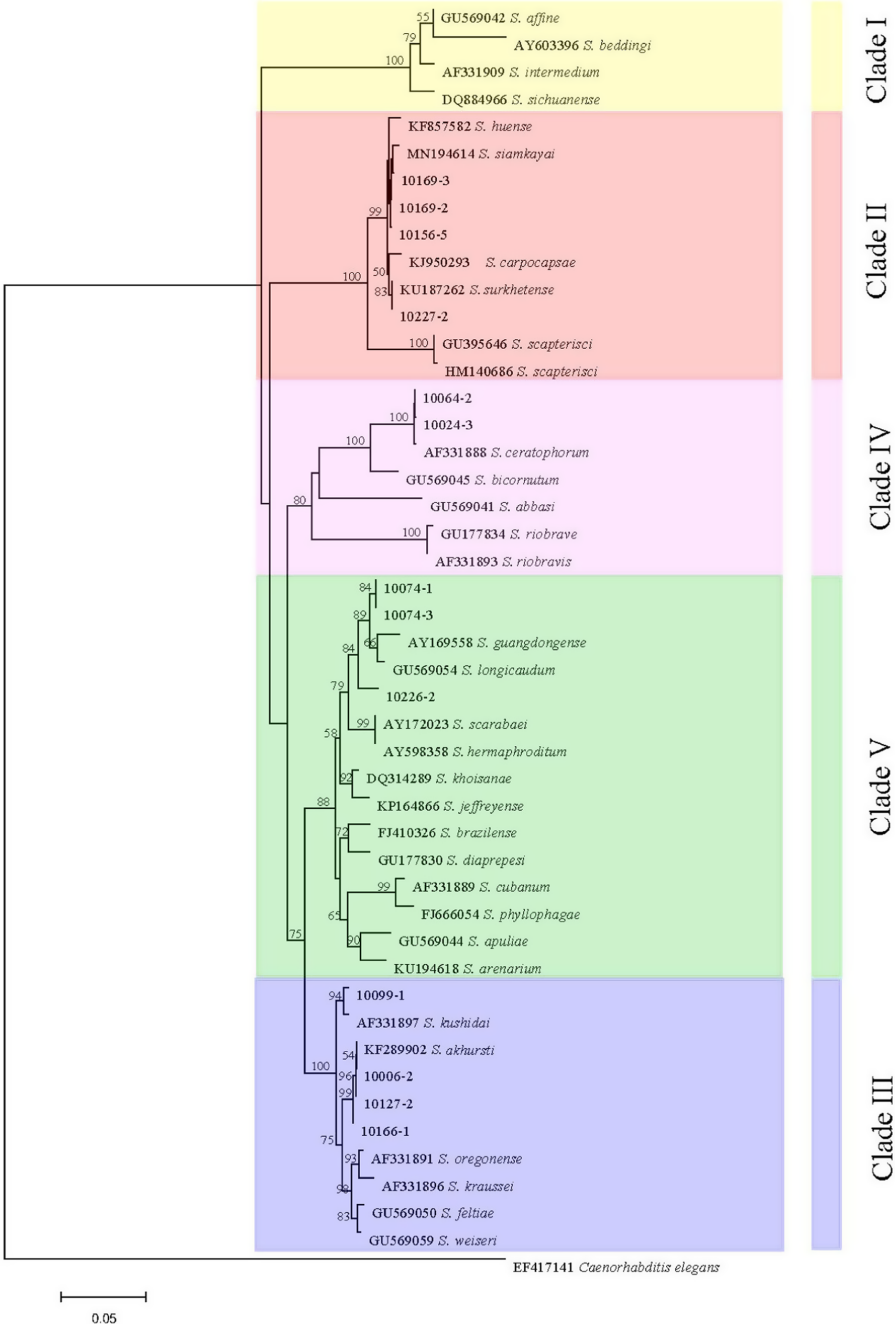
Neighbor-Joining tree of the *Steinernema* populations. The tree was based on 28S rDNA data and Kimura 2-parameter model. Numbers on branches represent bootstrap support (>50%) based on 1,000 replicates. Scale represents K2P genetic distance.

**Figure 4:**
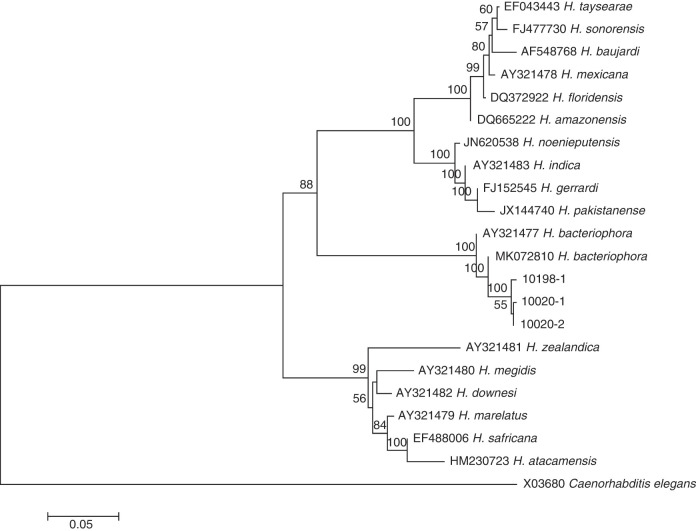
Neighbor-Joining tree of the *Heterorhabditis* populations. The tree was based on ITS rDNA data and Kimura 2-parameter model. Numbers on branches represent bootstrap support (>50%) based on 1,000 replicates. Scale represents K2P genetic distance.

**Figure 5:**
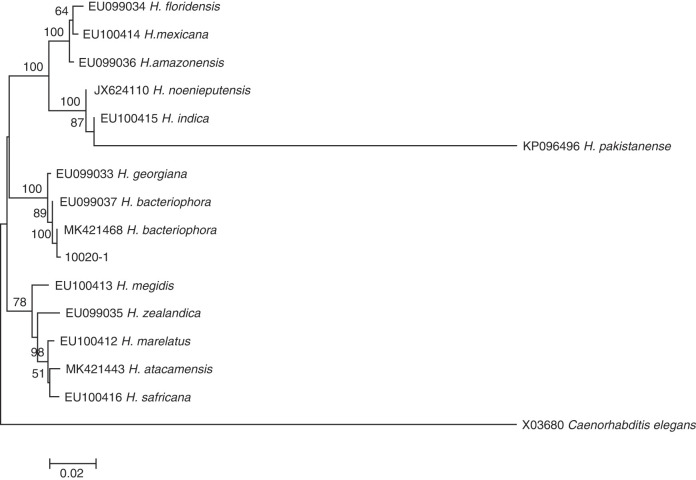
Neighbor-Joining tree of the *Heterorhabditis* populations. The tree was based on 28S rDNA data and Kimura 2-parameter model. Numbers on branches represent bootstrap support (>50%) based on 1,000 replicates. Scale represents K2P genetic distance.

**Figure 6:**
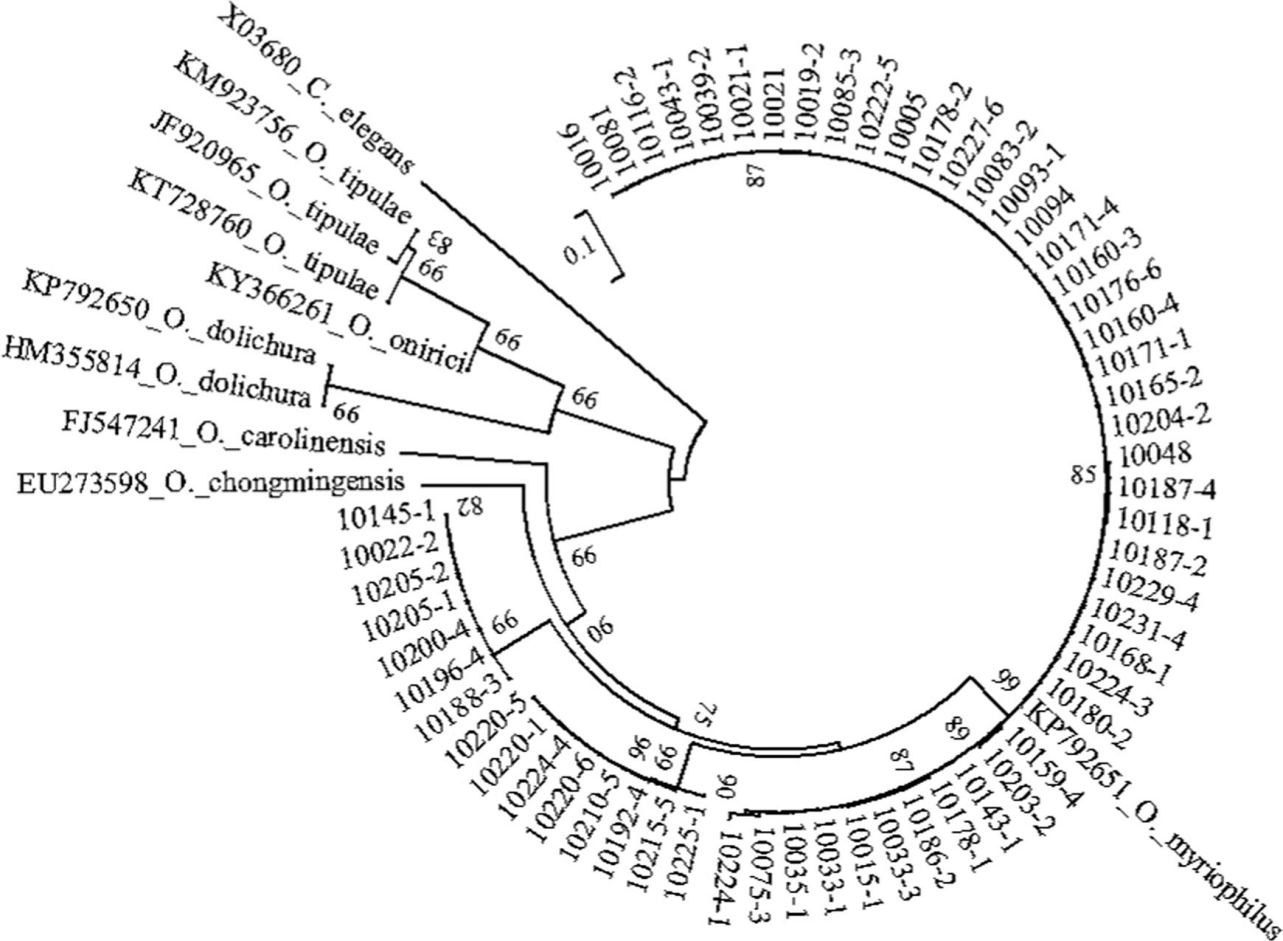
Neighbor-Joining tree of the *Oscheius* populations. The tree was based on ITS rDNA data and Kimura 2-parameter model. Numbers on branches represent bootstrap support (>50%) based on 1,000 replicates. Scale represents K2P genetic distance.

**Figure 7:**
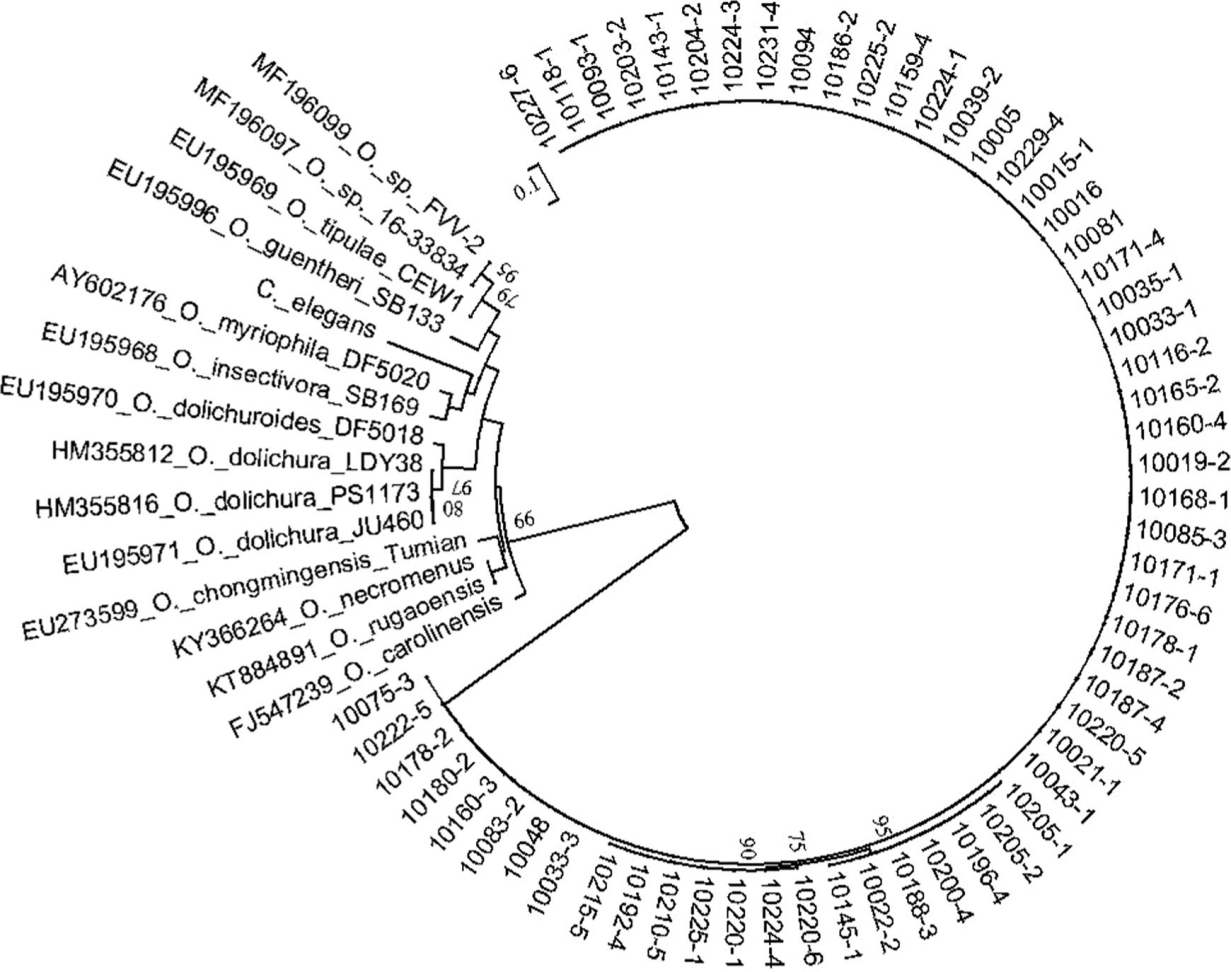
Neighbor-Joining tree of the *Oscheius* populations. The tree was based on 28S rDNA data and Kimura 2-parameter model. Numbers on branches represent bootstrap support (>50%) based on 1,000 replicates. Scale represents K2P genetic distance.

The studied isolates were identified and characterized based on ITS, 28S, and COI genes of the rDNA. DNA sequences were blasted against the NCBI database in GenBank, and all isolated species were found to belong to the genera of *Steinernema*, *Heterorhabditis*, and *Oscheius*. Their prevalence was shown in Figure 1B. BLAST analysis showed that, of the 75 amplified sequences, 13 matched with various species of EPNs in GenBank belonging to *Steinernema*, three belonging to *Heterorhabditis* and 58 belonging to *Oscheius* (Figs. 2–7). For all 789 samples, the prevalence of *Heterorhabditis* and *Steinernema* was around 2.03%, and 7.35% for *Oscheius*. The forest and farmland and waterside had higher prevalence than grassland and the edge of farmland. The EPNs from *Steinernema* were detected in all vegetation covers whereas *Heterorhabditis* was detected only in cropland. Forest and waterside had higher prevalence of *Oscheius.* Only one species *H. bacteriophora* from the genus of *Heterorhabditis*, and five known species from *Steinernema* were detected in these samples, including *S. akhursti*, *S. ceratophorum*, *S. everestense*, *S. siamkayai*, *S. surkhetense* and four unknown species (Table 2). The pH of soil samples with the presence of *Heterorhabditis* and *Steinernema* ranged from 4.51 in forest to 7.99 in cropland. The cation exchange capacity (CEC) in these soil samples ranged from 0.23 to 23.91 cmol/kg. With regard to the total nitrogen (TN), it ranged from 0.91 g/kg in cropland to 40.27 g/kg in cropland whereas soil organic matter (SOC) content ranged from 5.6 g/kg in forest to 74.9 g/kg in forest (Table 1).

**Table 2.** The list of indigenous species in the genus of *Heterorhabditis* and *Steinernema* detected from Yunnan province, China Species and soil physicochemical parameters of the corresponding habitats.

	Soil No.	Habitat	Longitude	Latitude	Altitude (m)	pH	SOC (g/kg)	TN (g/kg)	CEC (cmol/kg)	Clay	Sand	Silt
*H. bacteriophora*	10020-1	cropland	24.856385	103.366102	1850	6.55	28.5	1.55	12.23	47	25	28
	10020-2	cropland	24.856385	103.366102	1850	6.92	15.8	0.91	8.34	47	25	28
	10198-1	cropland	26.1089	99.9999	2055	7.65	64.4	3.26	21.54	36	39	25
*S. akhursti*	10006-2	forest	25.51231	100.2601	2482	4.51	56	1.86	13.94	22	51	27
*S. akhursti*	10127-2	forest	24.16796	102.6275	1818	4.55	29.6	17.17	1.56	26	36	38
*S. ceratophorum*	10024-3	forest	24.93598	103.5364	1837	5.17	24.2	1.35	13.96	47	25	28
*S. ceratophorum*	10064-2	cropland	23.80083	103.6483	1456	7.84	19.5	1.19	23.91	36	39	25
*Steinernema* sp. Sun 1	10074-1	forest	23.65063	102.7021	1391	6.84	21.6	1.17	7.77	36	39	25
*Steinernema* sp. Sun 1	10074-3	forest	23.65063	102.7021	1391	6.57	74.9	3.58	18.52	36	39	25
*Steinernema* sp. Sun 2	10099-1	forest	24.07291	101.9257	1861	6.06	40.6	23.57	1.52	36	39	25
*S. siamkayai*	10156-5	waterside	23.21695	101.1314	1117	6.94	24.7	14.35	1.39	22	51	27
*S. everestense*	10166-1	cropland	23.65539	100.0582	1820	5.06	69.4	40.27	3.34	31	36	33
*Steinernema* sp. Sun 3	10169-2	grassland	24.17514	99.98811	1124	4.83	18	10.45	1.06	43	28	29
*Steinernema* sp. Sun 3	10169-3	grassland	24.17514	99.98811	1124	4.93	42	24.34	2.11	43	28	29
*Steinernema* sp. Sun 4	10226-2	forest	24.4748	97.7456	1096	6.24	5.64	3.27	0.23	39	34	27
*S. surkhetense*	10227-2	cropland	24.1877	97.8166	949	7.99	6.25	3.62	0.39	43	28	29

**Note:** CEC: cation exchange capacity; SOC: soil organic matter, TN: total nitrogen.

Phylogenetic analysis of the 28S rDNA and ITS rDNA sequence data showed that58 collected isolates were associated with *O. myriophilus*. The length of the ITS gene for *Oscheius* isolate was 816 bps, which had 99% similarities and 100% of query coverage with the ITS sequences of *O. myriophilus* (MT328660). The phylogenetic tree based on ITS showed that *Oscheius* isolates detected from Yunnan province formed a monophyletic group, and this group of *Oscheius* was placed in a single clade (Fig. 6). The length of the 28S rDNA for *Oscheius* isolate was 867 bps. Then BLAST analysis of the 28S rDNA for the Yunnan population attributes to 99% similarities and 100% of query coverage with the 28S sequences of *O. myriophilus* (MG742109). The phylogenetic tree reconstructed based on 28S rDNA sequences showed that *Oscheius* YN formed a monophyletic group with *O. myriophilus.* Figure 7 shows that the bootstrap value of support for the corresponding clade countering *O. myriophilus* and other isolates was 99%. Obviously, *O. myriophilus* is one of the most widely distributed EPNs in Yunnan province that will require further study.

### Virulence of different EPNs populations against *S. litura*

**Figure 8:**
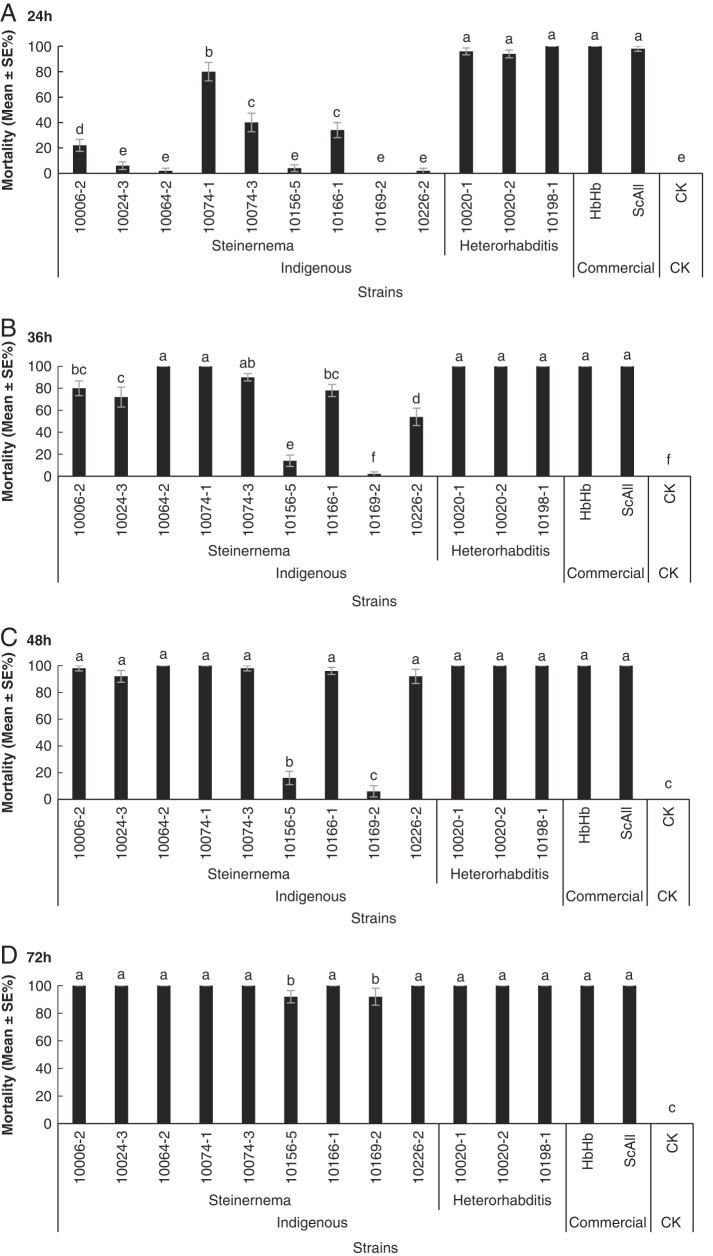
Mortality rates (mean ± SE%) of *Spodoptera litura* larvae treated 24, 36, 48, and 72 hr by different populations of entomopathogenic nematodes at 100 infective juveniles per larva. HbHb: *Heterorhabditis bacteriophora*; Sc All: *Steinernema carpocapsae*; CK: Control. Bars with different letters represent significantly different means (SE) at *p* ≤ 0.05 among all treatments via the post hoc Duncan’s test.

All tested EPNs were capable of infecting and killing *S. litura*. However, the virulence varied among the different isolates. The mortality of *S. litura* exposed to *Steinernema* and *Heterorhabditis* isolates at the rate of 100 IJs/larvae was presented in Figure 8. The mortality of *S. litura* at 24 and 48 h after *Steinernema* and *Heterorhabditis* application was significantly different among the tested isolates (24 h: *F*= 145, df = 14,139, *p* <  0.001; and 48 h: *F* = 243, df = 14,139, *p* < 0.001, respectively). Five tested EPNs isolates, *H. bacteriophora* 10020-1, *H. bacteriophora* 10020-2, *H. bacteriophora* 10198-1, *S. carpocapsae* All and *H. bacteriophora* HbHb caused approximately 100% larval mortality of *S. litura* after 24 hr exposure. Two isolates *S. siamkayai* 10156-5 and *S. siamkayai* 10169-2 had relatively low mortality of *S. litura* after 48 hr exposure whereas the mortality in these two treatments reached 90% after 72 hr exposure. The efficacy of other indigenous isolates was close to those of commercial populations, indicating that indigenous EPNs could be used as biological control agents to control *S. litura* in Asia-Pacific region.

**Figure 9:**
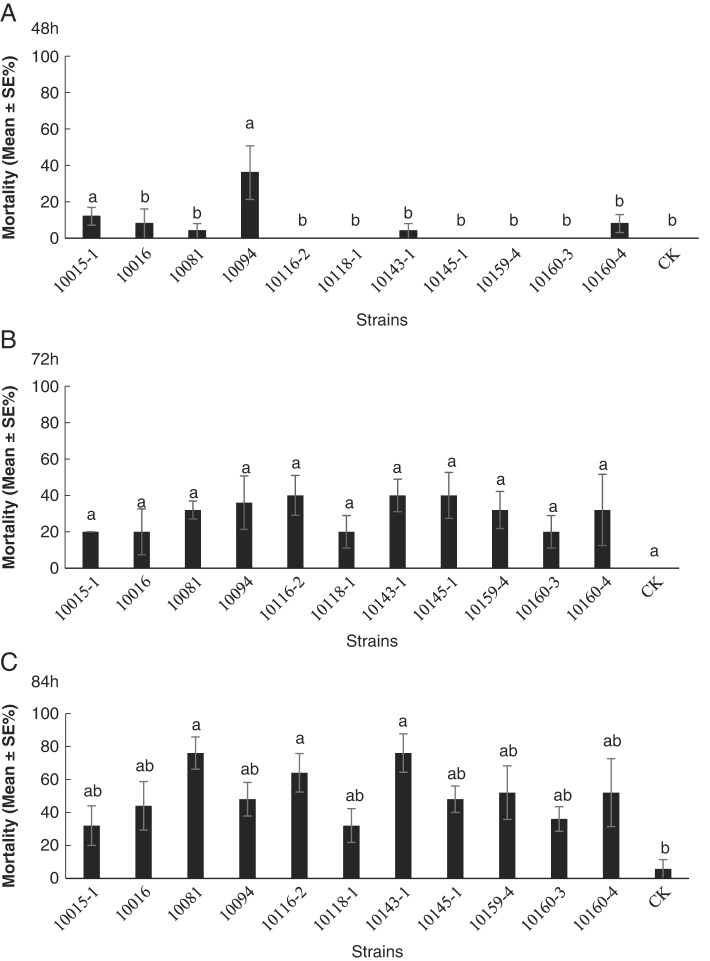
Mortality rates (mean ± SE%) of *Spodoptera litura* larvae treated 48, 72, 84 hr by different populations of *Oscheius* at 100 infective juveniles per larva. CK: Control. Bars with different letters represent significantly different means (SE) at *p* ≤ 0.05 among all treatments via the post hoc Duncan’s test.

The mortality of *S. litura* exposed to *Oscheius* isolates was presented in Figure 9. Three of those tested *Oscheius* isolates, *O. myriophilus* 10081, *O. myriophilus* 10116-2, *O. myriophilus* 10143-1 caused approximately 60–80% larval mortality of *S. litura* after 84 h exposure (Fig. 9C), and reached the significant difference as compared with the control group.

## Discussion

We performed an intensive survey of EPN across Yunnan province, which is a biodiversity hotspot in China. In total, we collected 789 soil samples at 232 sites. Eventually, 13 isolates belonging to five *Steinernema* species were recovered from diverse habitats. Additionally, three isolates belonging to *Heterorhabditis* were identified as *H. bacteriophora.* Moreover, 58 isolates belonging to *Oscheius* were confirmed as EPNs using Koch’s postulates via the second inoculation. This is the first intensive EPN survey across the entire region of Yunnan province. The prevalence of *Heterorhabditis* and *Steinernema* was around 2.03%, and that of *Oscheius* was 7.35%, which was consistent with reports from the other regions. Globally, the prevalence of EPNs in soil samples was reported to range from 0.7 to 50% (Benseddiq et al., 2020; Hominick, 2002) though specific sampling methods with repeated extraction technique may exceed this range (Abd-Elgawad, 2020). Merits and demerits of nematode-sampling and extraction methods were recently reviewed (Abd-Elgawad, 2021).

In the present survey, *H. bacteriophora* was mainly in neutral and basic soils associated with cropland (Table 2) whereas *Steinernema* spp. were presented in the soil with a pH ranging from 4.51 to 7.99 associated with forest (43.75% of the total positive sites), suggesting that *Steinernema* spp. had adapted to the acid soil (Kanga et al., 2012). These results are consistent with other findings of the previous studies where *Steinernema* spp. can occur in the acid soil (Campos-Herrera et al., 2019). In general, the soil clay content was inversely related to the presence of EPNs (Ardpairin et al., 2020; Mracek et al., 2005). Likewise, the isolated of *Heterorhabditis* or *Steinernema* were found in the soil with the soil clay content lower than 50 in our study. Other physicochemical properties such as SOC (6.25–64 g/kg), TN (0.91–23.57 g/kg), and CEC (0.39–23.91) showed great variations among the sites with the presence of *Heterorhabditis* or *Steinernema* populations, indicating that their distributions are not related to these properties. This is in accordance with the findings of Campos-Herrera et al. (2019).

Currently, species belonging to the genus *Oscheius* have been reported as EPNs (Castro-Ortega et al., 2020). We found that EPNs in the genus *Oscheius* are widely distributed in Yunnan province, especially in the habitats of forest and waterside. Koch’s postulates were performed to test the virulence of the isolated *Oscheius,* and 58 isolates were confirmed as EPNs. Although *Oscheius* generally need longer time to kill insect pests than in *Steinernema* and *Heterorhabditis* (Goda et al., 2020; Ye et al., 2018). Considering their wide distribution, *Oscheius* could play a crucial role in regulating soil insect populations, especially in regions where *Steinernema* and *Heterorhabditis* are absent. The results obtained in our research highlight the potential of EPNs in *Oscheius* to be used as biological control agent in the future.

The five known *Steinernema* species including *S. akhursti*, *S. ceratophorum*, *S. everestense*, *S. siamkayai*, *S. surkhetense* and four unknown *Steinernema* species were found in our intensive survey. *Steinernema akhursti* was first recovered from soil samples collected from Zhongdian county, Diqing district in the northwest part of Yunnan province (Qiu et al., 2005b). *Steinernema ceratophorum* was first isolated from Liaoning Province, north-east China in 1994 (Jian et al., 1997). *Steinernema everestense* was first discovered and described in Nepal (Spiridonov et al., 2011), and this time it was first recorded in China. *Steinernema siamkayai* was first discovered and described in Thailand (Stock et al., 1998), and it was first recorded in China. *Steinernema surkhetense* was first discovered and described in Nepal (Khatri-Chhetri et al., 2011) and it was first recorded in China. However, *S. xueshanense* (Mracek et al., 2009), *S. beddingi* (Qiu et al., 2005c), and *S. pui* (Qiu et al., 2011) that were found in previous studies in Yunnan province were not detected in our study.

The phylogenetic tree based on Neighbor-Joining proposed a reasonably good resolution among the distal clades. Our wide-ranging analyses of the phylogeny constructed on the ITS region provides a novel vision into the interactions between groups and species in Steinernematids. The results support the same evolutionary lines previously found by Stock et al. (2001) that were based on the partial sequence of the 28S rDNA. In both analyses, five major clades were perceived, although the associations among the clades were not strong when using ITS sequences compared to 28S rDNA. Unfortunately, we could not build a phylogenetic tree based on COI data of all the isolates of *Steinernema*, *Heterorhabditis*, and *Oscheius* detected in Yunnan province since the dearth of available public database.

All molecular systematic approaches to find out the relationships among species populations of *Heterorhabditis* have been done using the ITS rDNA and 28S rDNA. Three collected *Heterorhabditis* isolates were associated with only one species of *H. bacteriophora*. According to Shapiro-Ilan et al. (2008), there was close relationship between *H. bacteriophora* and *H. georgiana* and this relationship was also shown in ourphylogram. The overall topology of our phylogenetic tree is consistent with the previous studies (Adams et al., 2006).

In our study, 14 EPN isolates in the genera *Heterorhabditis* and *Steinernema* were evaluated for their virulence against the 5th instar larvae of *S. litura* and two commercial isolates were included as a positive control. These isolates varied in their virulence against the 5th instars of *S. litura*. *Heterorhabditis* sp. 10020-1, 10020-2, and 10198-1 can quickly kill *S. litura* larvae compared to other indigenous EPN isolates within 24 hr. All tested *Heterorhabditis* and *Steinernema* isolates caused over 90% mortality of *S. litura* after 72 hr exposure. Seven indigenous EPN populations in Philippines including *S. abbasi*, *S. minutum*, *S. tami*, and *H. indica* were isolated from 279 soil samples. Of them, *S. abbasit* and *H. indica* heir showed higher virulence against the insect pest *S. litura* than EPN populations of *S. minutum*, *S. tami* (Caoili et al., 2018). Ten indigenous populations of *H. indica* were evaluated for their differential infectivity against insect pest *S. litura,* and all populations showed high virulence but only one population were able to produce infective juveniles and the rest failed to do so (Gokte-Narkhedkar et al., 2019). In another study, five indigenous EPN populations in China showed various virulence against *S. litura*. Of them 64-2 including in the preset study showed high higher virulence to 2th, 3th, and 4th star larvae of *S. litura* (Yan et al., 2020). In addition, we tested the virulence of the *Oscheius* populations against *S. litura.* However, only part of *Oscheius* populations were employed for virulence bioassay since some of *Oscheius* populations cannot be successfully maintained through several cycles of reinfections of last-instar larvae of *G. mellonella*.

In future studies on virulence, different populations of EPNs will be used to evaluate the virulence of the host insect in different instars, which will be important as multiple stages of the target pest may be available to the EPNs during field application.

Actually, the present study only showed innate virulence of the EPNs under laboratory conditions. Will the indigenous populations show higher biocontrol efficacy in the field given that they may be adapted to local conditions – more so than commercial populations? Native species are well adapted to local climatic conditions and are generally considered the best choice for natural enemy species. Priority has been given to the use of native species in order to reduce possible environmental risks (Odendaal et al., 2016). We need more field work to compare the pathogenicity of indigenous and commercial populations, and even the persistence in the local soil circumstances. In addition, we did not detect the isolates of *S. feltiae* and *S. carpocapsae* in our survey, which are used as commercial biological agents. More intensive survey perhaps needs to be conducted in Yunnan province to find more beneficial nematodes for future field application. The indigenous EPN isolates in the genera *Steinernema* and *Heterorhabditis* showed great potential virulence for *S. litura*.

Here, it is worthwhile to discuss the status of *Oscheius* as EPNs. Most of *Oscheius* populations might not be regarded as EPNs according to the criteria set by killing the target insect within 48 hr (Zhang et al., 2019). Similarly, many EPN populations from the genus of *Steinernema* and *Heterorhabditis* also cannot be regarded as EPNs given that they cannot kill the pest insects within 48 hr. Apparently the present concept of EPNs is named only based on their great biocontrol potential with regard to their virulence. Nevertheless, it might not be reasonable to define as EPNs only with mortality time within 48 hr. In fact, the populations from *Oscheius* were able to kill target pest insect finally when they were isolated, it cannot be excluded that these populations might maintain their virulence in the natural habitats and the losing of virulence in lab culture might not occur. Moreover, considering the higher rate of positive in the soil sample in the present survey, the populations in *Oscheius* i.e. *O. myriophilus* might play a crucial role in regulating the soil-dwelling insect population in this area, especially in the area with the absence of *Steinernema* and *Heterorhabditis*. Further study will be needed to elucidate their ecological role for pest insect control. Hence, some *Oscheius* populations might be regarded as an important biological control factor once their virulence is confirmed. Meanwhile, their characteristic as scavenger also is of quite importance to maintain their population (Blanco-Pérez et al., 2017, 2019).

EPNs serve as important population regulators of insect species with soil-dwelling stages, and have been applied as biological control agents for a variety of economically important pest insects, i.e. *S. litura.* The present survey successfully obtained 10 EPN species including one known species in *Heterorhabditis*, and five known and four known species in *Steinernema*. Unlike other studies, besides those species in *Steinernema* and *Heterorhabditis*, we also investigated the distribution and diversity of *Oscheius.* Totally, 58 populations from *Oscheius*, most of them belonging to the species of *O. myriophilus*, were showed virulence against *G. mellonella* when they were isolated. *Oscheius* spp. might play a role for pest insect control, especially in the area with the absence of *Steinernema* and *Heterorhabditis*. What is extent of contribution of *Steinernema*, *Heterorhabditis*, and *Oscheius* to target pest control in the really natural field condition is needed to elucidate in the further study. Moreover, compared other biological control agents, i.e. fungi, bacteria, virus, the proportion of pest insect mortality by EPNs also needed to be addressed across biogeographic and climatic regions. With the intense studies on biology and ecology of these isolated indigenous nematodes as well as improved application technologies (Bruno et al., 2020), some indigenous nematodes with great infectivity in combination with commercial EPNs or other biological control agents such as entomopathogenic fungi can be used for integrated pest control, i.e. *S. litura.*
